# Digital Health Technologies Respond to the COVID-19 Pandemic In a Tertiary Hospital in China: Development and Usability Study

**DOI:** 10.2196/24505

**Published:** 2020-11-24

**Authors:** Wanmin Lian, Li Wen, Qiru Zhou, Weijie Zhu, Wenzhou Duan, Xiongzhi Xiao, Florence Mhungu, Wenchen Huang, Chongchong Li, Weibin Cheng, Junzhang Tian

**Affiliations:** 1 Information Department Guangdong Second Provincial General Hospital Guangzhou China; 2 Internet Hospital Guangdong Second Provincial General Hospital Guangzhou China; 3 Department of Toxicology School of Public Health Southern Medical University Guangzhou China; 4 Dott Medical Co Ltd Shenzhen China; 5 Beijing Rxthinking Technology Co Ltd Beijing China; 6 Institute for Healthcare Artificial Intelligence Application Guangdong Second Provincial General Hospital Guangzhou China

**Keywords:** Internet hospital, COVID-19, automated screening, symptom, monitoring, web-based consultation, psychological support, emergency, digital health, hospital, China, screening

## Abstract

**Background:**

The outbreak of COVID-19 has caused a continuing global pandemic. Hospitals are integral to the control and prevention of COVID-19; however, they are facing numerous challenges during the epidemic.

**Objective:**

Our study aimed to introduce the practical experience of the design and implementation of a web-based COVID-19 service platform at a tertiary hospital in China as well as the preliminary results of the implementation.

**Methods:**

The web-based COVID-19 service platform was deployed within the health care system of the Guangdong Second Provincial General Hospital and Internet Hospital; the function of the platform was to provide web-based medical services for both members of the public and lay health care workers. The focal functions of this system included automated COVID-19 screening, related symptom monitoring, web-based consultation, and psychological support; it also served as a COVID-19 knowledge hub. The design and process of each function are introduced. The usage data for the platform service were collected and are represented by three periods: the pre-epidemic period (December 22, 2019, to January 22, 2020, 32 days), the controlled period (January 23 to March 31, 2020, 69 days), and the postepidemic period (April 1 to June 30, 2020, 91 days).

**Results:**

By the end of June 2020, 96,642 people had used the automated COVID-19 screening and symptom monitoring systems 161,884 and 7,795,194 times, respectively. The number of general web-based consultation services per day increased from 30 visits in the pre-epidemic period to 122 visits during the controlled period, then dropped to 73 visits in the postepidemic period. The psychological counseling program served 636 clients during the epidemic period. For people who used the automated COVID-19 screening service, 160,916 (99.40%) of the total users were classified in the no risk category. 464 (0.29%) of the people were categorized as medium to high risk, and 12 people (0.01%) were recommended for further COVID-19 testing and treatment. Among the 96,642 individuals who used the COVID-19 related symptoms monitoring service, 6696 (6.93%) were symptomatic at some point during the monitoring period. Fever was the most frequently reported symptom, with 2684/6696 symptomatic people (40.1%) having had this symptom. Cough and sore throat were also relatively frequently reported by the 6696 symptomatic users (1657 people, 24.7%, and 1622 people, 24.2%, respectively).

**Conclusions:**

The web-based COVID-19 service platform implemented at a tertiary hospital in China is exhibited to be a role model for using digital health technologies to respond to the COVID-19 pandemic. The digital solutions of automated COVID-19 screening, daily symptom monitoring, web-based care, and knowledge propagation have plausible acceptability and feasibility for complementing offline hospital services and facilitating disease control and prevention.

## Introduction

The outbreak of COVID-19 has caused an ongoing global pandemic that is presently affecting over 37 million people worldwide. Hospitals have been at the center of the COVID-19 control and prevention effort while facing many challenges during the epidemic. The surging demands of health care for COVID-19 screening and treatment have overwhelmed the medical system [1,2], and the lack of proper personal protective equipment for medical staff is causing nosocomial infection concerns [3,4]. Additionally, maintaining routine care services such as chronic condition care and emergency outpatient visits while suspending general outpatient visits during the epidemic has placed stress on hospitals [5-7].

The Guangdong Second Provincial General Hospital (GD2H) is a large-scale tertiary hospital located in Guangzhou, China, and it is renowned for its emergency medical rescue and smart hospital services. GD2H established the first internet hospital in China in October 2014, and it is a pioneering center in the exploration of smart hospitals [8]. In 2019, the internet hospital was upgraded with a new application equipped with over 20 digital health technologies, including artificial intelligence (AI) physician services, distance electrocardiogram diagnosis, a prescription circulation platform, and a medical imaging diagnosis system. These digital technologies help shift tasks from the hospital to the community. Based on the internet hospital, GD2H established a tiered health care delivery system that provides professional medical care support for lay health care workers in 2377 poor villages in Guangdong, China. In the application of 5G technology, GD2H took the lead in establishing the 5G distance surgery practice in Guangdong Province. Meanwhile, as the first provincial emergency hospital in China and the seventh World Health Organization Emergency Medical Team, GD2H was one of the major designated COVID-19 treatment centers in response to the COVID-19 outbreak in Guangdong, China.

Digital health solutions, including internet hospitals, have been reported to facilitate epidemic control measures such as contact tracing and prehospital triaging while providing web-based medical care [9-14]. There has been a surge in the establishment of internet hospitals during the COVID-19 pandemic. A total of 213 new internet hospitals (compared to 362 by the end of 2019) were established between January and June 2020 in China. Over 47,000 physicians voluntarily provide medical care services on Haodf.com (a private internet hospital platform) [15]. However, less information has been documented concerning the implementation perspective of these technologies in hospitals [16]. In this paper, we introduce the practical experience of design and implementation as well as the preliminary results of an internet hospital–based web-based COVID-19 service platform; its functions include automated COVID-19 screening, monitoring of related symptoms, and web-based care services, and it additionally serves as a knowledge hub.

## Methods

### Service Framework

**Figure 1:**
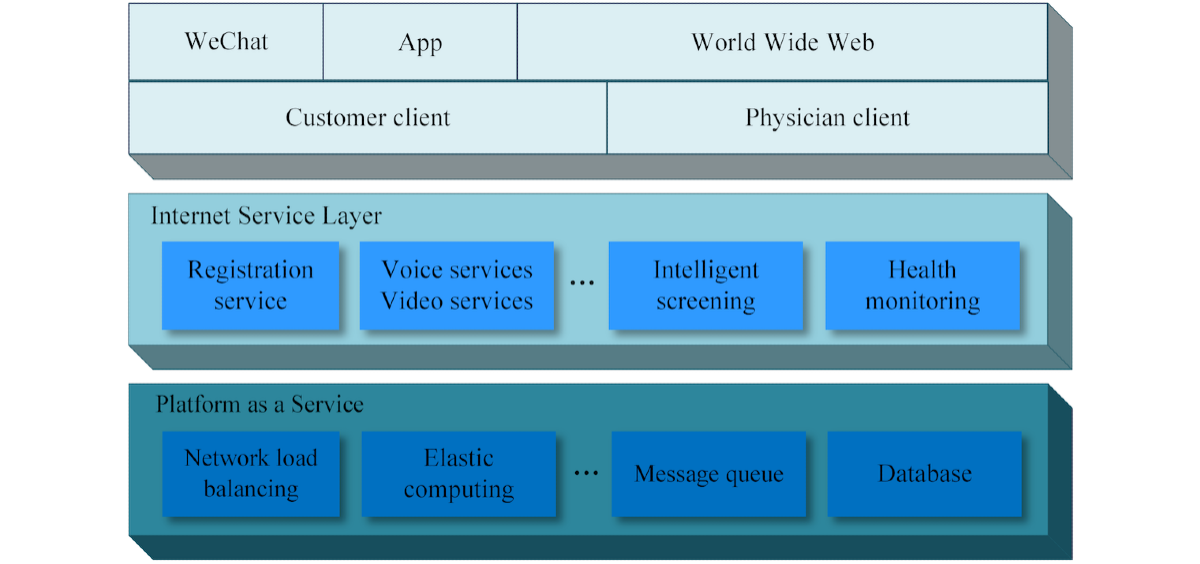
The web-based medical service framework.

The web-based COVID-19 platform was deployed at the GD2H Internet Hospital [8], which provides web-based medical services for both public individuals (ie, customer clients) and lay health care workers (ie, physician clients, rural health care personnel without formal medical training). The service framework is shown in Figure 1. The upper layer shows the portal of the service, which includes WeChat (a popular social media platform in China, similar to Facebook and Twitter), a smartphone app (developed by the GD2H), a website, and target users. The main functions include automated COVID-19 screening, monitoring of related symptoms, web-based consultation, psychological support, and COVID-19 knowledge dissemination. Modern information technologies such as the Internet of Things, big data, and AI were featured in the services platform.

### Automated COVID-19 Screening

**Figure 2:**
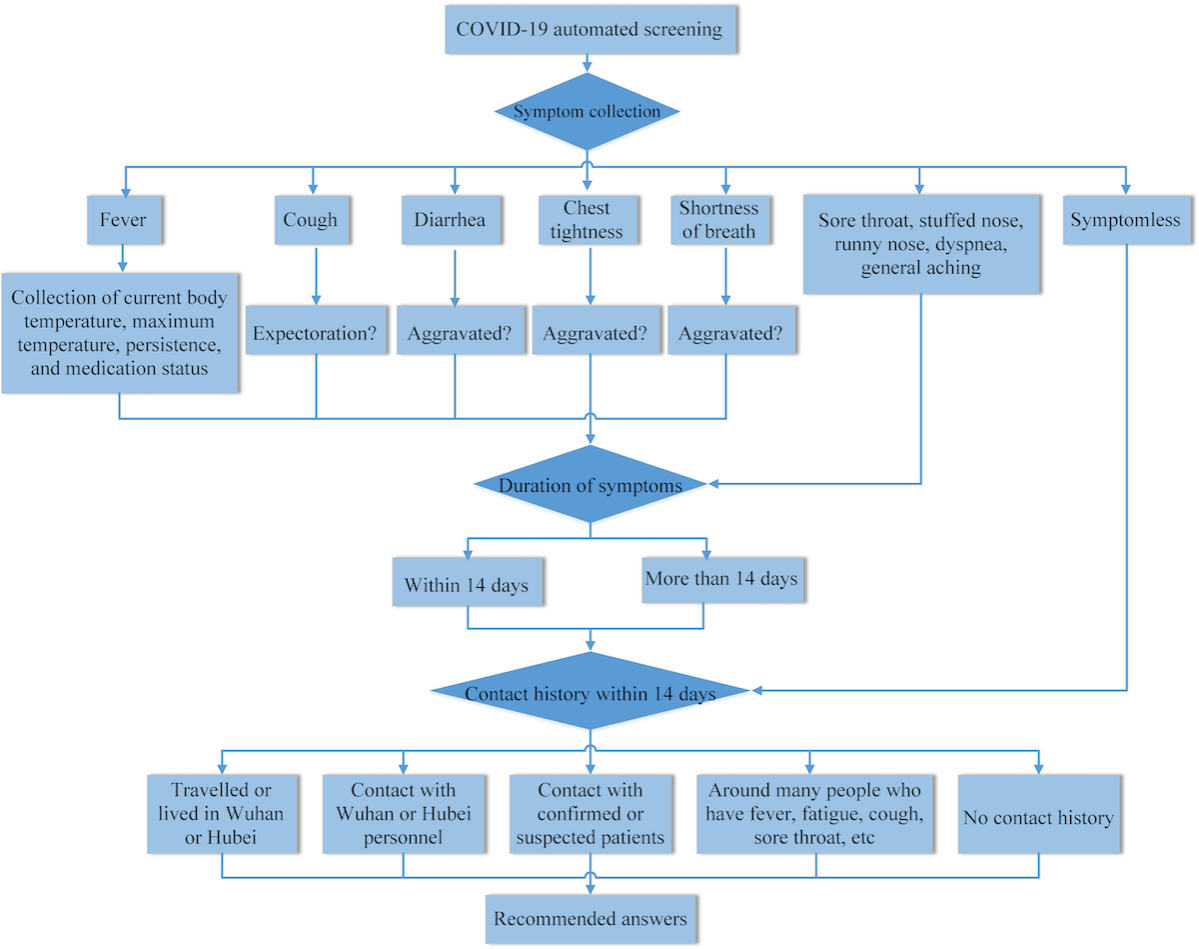
Flowchart for automated COVID-19 screening.

This service functions as a forward triage strategy that enables patients to be efficiently screened before they arrive at the hospital emergency department; this strategy protects patients, clinicians, and the community from exposure. Automated screening algorithms were designed based on a decision tree that classified patients according to their symptoms, travel history, and exposure to COVID-19. Clients who met the epidemiological suspected criteria were then transferred to a web-based physician consultation for further screening and care. Inclusion of symptoms, determination of duration, and contact history were based on the COVID-19 Diagnosis and Treatment Protocol (trial version 7) issued by the National Health Commission of the People’s Republic of China [17]. Based on the report, individuals were classified as no risk, low risk, medium to high risk, and high risk. The definitions of the risk categories are provided in Multimedia Appendix 1. The process of automated COVID-19 screening is shown in Figure 2.

### Monitoring of COVID-19–Related Symptoms

This service was designed to facilitate individuals’ symptom self-management and staff health status management by organizations. The symptom monitoring protocol was based on the China Center for Disease Control and Prevention guideline for monitoring of close contacts of COVID-19 cases [18]. Typical COVID-19–related symptoms, such as fever, sore throat, and fatigue, were collected in a structured way twice daily and self-reported through the app portal. A dashboard displayed visual graphics to show the changes in symptoms for users and physicians. Physicians of the internet hospital were then alerted of any abnormal statuses in real time, and the internet hospital physician would then reach out and provide guidance to the client for risk assessment and treatment. The abnormal results that triggered the web-based consultation services included body temperature ≥38 ºC; body temperature ≥37.3 ºC and sore throat; and body temperature ≥37.3 ºC and fatigue.

**Figure 3:**
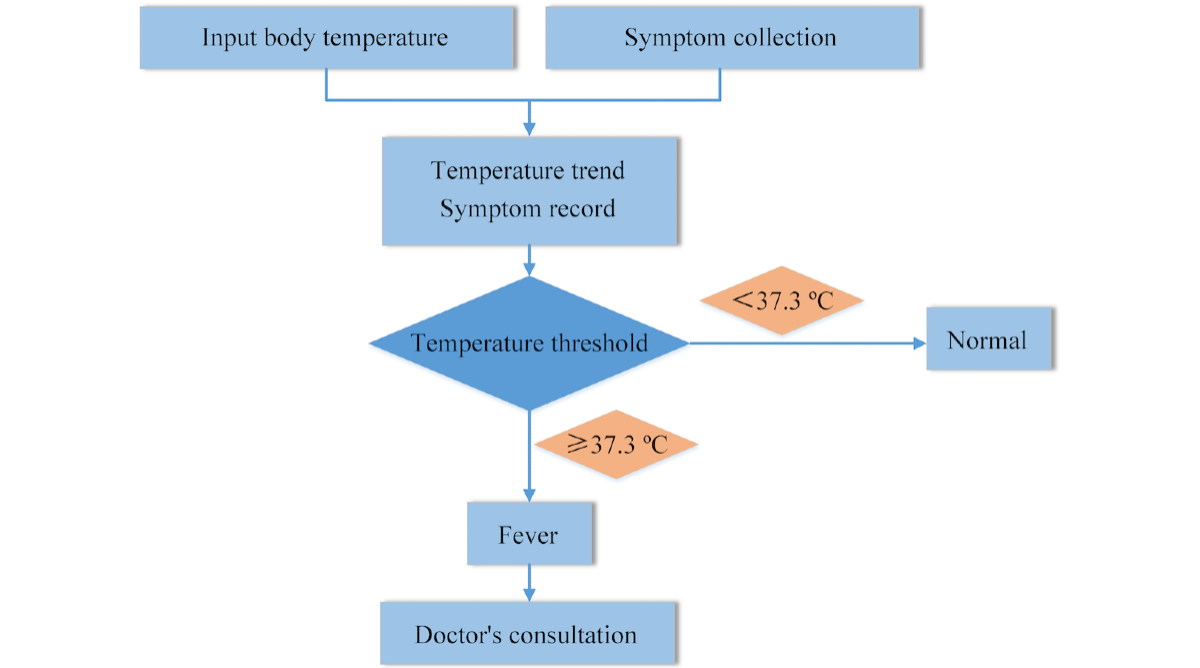
Flowchart for monitoring of COVID-19–related symptoms.

The process of COVID-19–related symptom monitoring is shown in Figure 3.

### Web-Based Consultation

Web-based consultation provides free general medical services and psychological counseling for the public through WeChat and the app, which links to the GD2H Internet Hospital. This service also provides specialized medical care support for lay health care workers who encounter problems while providing medical care in their villages and for primary care centers through a desktop system. Web-based consultation services can also be triggered when the results of automated COVID-19 screening or risk monitoring are abnormal.

General medical services were provided by 30 full-time professionally qualified physicians who were registered with the GD2H internet hospital. The physicians’ specialties covered internal medicine, surgery, traditional Chinese medicine, and rehabilitation. Psychological counseling services were provided by licensed psychiatrists; these services targeted not only the public and patients but also medical personnel to alleviate the psychological distress caused by the epidemic.

**Figure 4:**
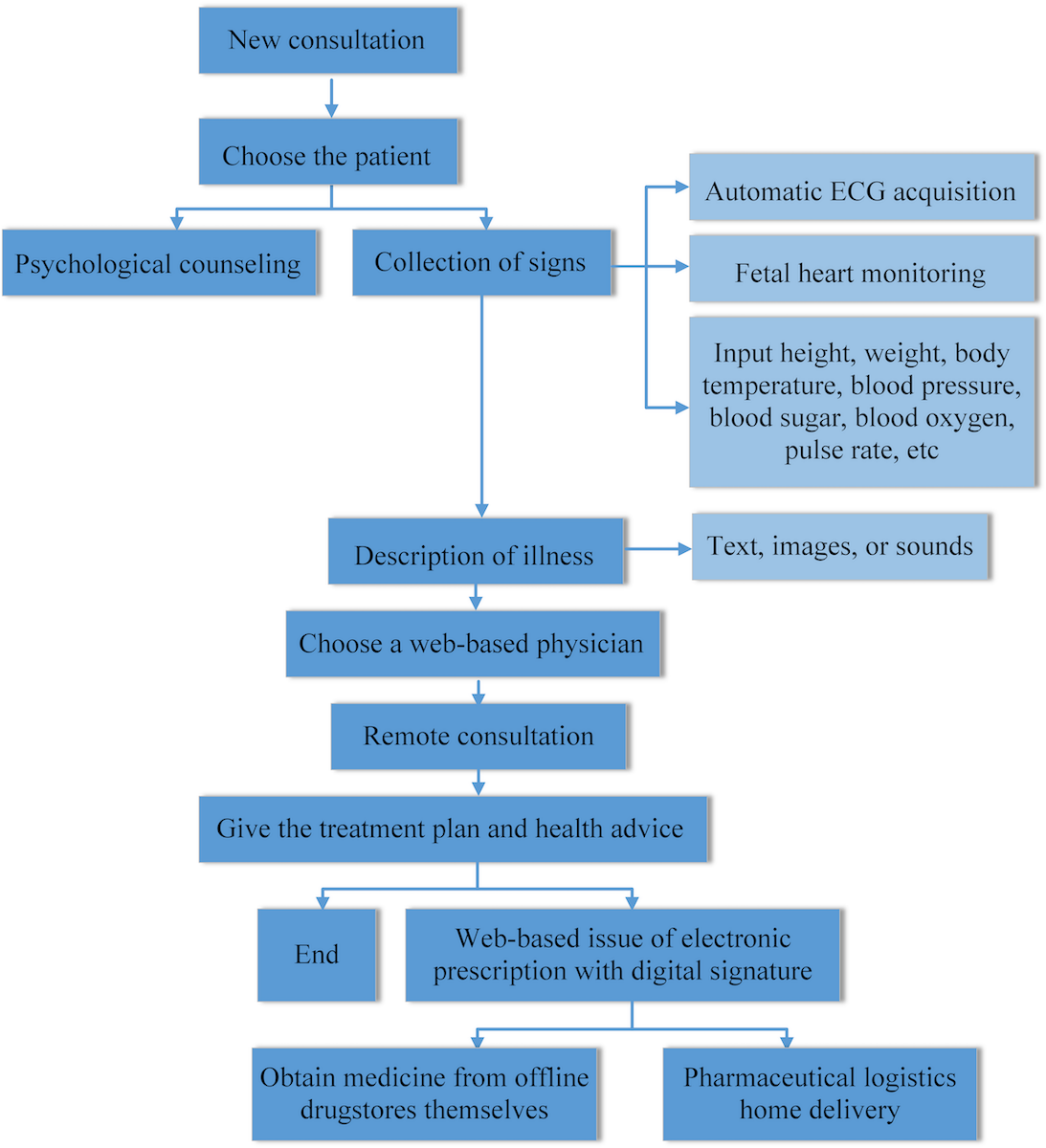
Flowchart of web-based consultation. ECG: electrocardiogram.

All physicians also received professional training on COVID-19 diagnosis and treatment. The web-based consultation service supports both text input and video consultation modes. The consultation interface enables users to upload materials including descriptions of illness, past medical history, symptoms, and test and examination results in various text, sound, and image formats; also, data uploaded by users are encrypted to protect privacy. Physicians prescribed authenticated digital prescriptions on the web, which were connected to a third-party drug distribution agency that provides home delivery service of drugs. The process of web-based COVID-19 consultation is shown in Figure 4.

### COVID-19 Knowledge Hub

The COVID-19 knowledge hub provides a COVID-19 encyclopedia to educate the public and help them understand the COVID-19 epidemic in a timely and comprehensive manner. COVID-19–related policies and prevention measures, treatment, and other information is appropriately updated. When new COVID-19 prevention and control information, such as updated guidelines and expert consensus, is officially released by the National Health Commission and the Chinese Society of Nutrition, it is pushed to the platform in infographic form for clients. The COVID-19 treatment module mainly provides detailed descriptions of use specifications, dosages, adverse reactions, and contraindications of common antiviral drugs, immunopotentiators, glucocorticoids, and antibacterial drugs. Traditional Chinese medicine, including specific prescription compositions, indications, and other information, is verified through practice by the traditional medicine and sports injury rehabilitation research team of GD2H.

### Data Collection and Analysis

Usage data of the platform were collected for this analysis. The automated COVID-19 screening, COVID-19–related symptom monitoring, and web-based consultation services have been in use since January 26, 2020. All data were collected up to June 30, 2020. On January 23, 2020, Wuhan was placed on official lockdown and the Guangdong Provincial Government declared the level one public health emergency response to COVID-19. By March 31, 2020, most areas in China were classified as low risk. A week later (April 8), Wuhan lifted its COVID-19 restrictions [19], which showed that the epidemic in China had been controlled. Based on these two time points, we divided the whole period into three sub-periods: the pre-epidemic period (before January 23), controlled period (January 23 to March 31), and postepidemic period (after March 31). Daily hospital service visit data (one month) were collected to compare the hospital service volumes between the preoutbreak and postoutbreak periods. All information was deidentified before analysis. The study was approved by the ethical review board of GD2H.

## Results

**Figure 5:**
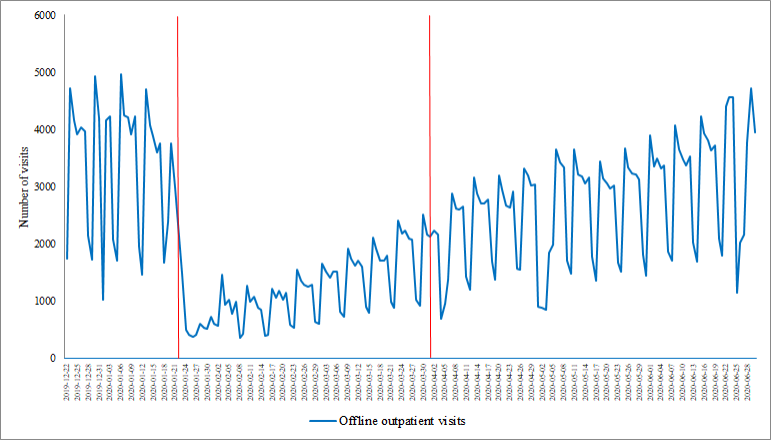
The trends of hospital outpatient visits from December 22, 2019, to June 30, 2020. The red lines denote the start of the controlled period and the end of the postepidemic period.

**Figure 6:**
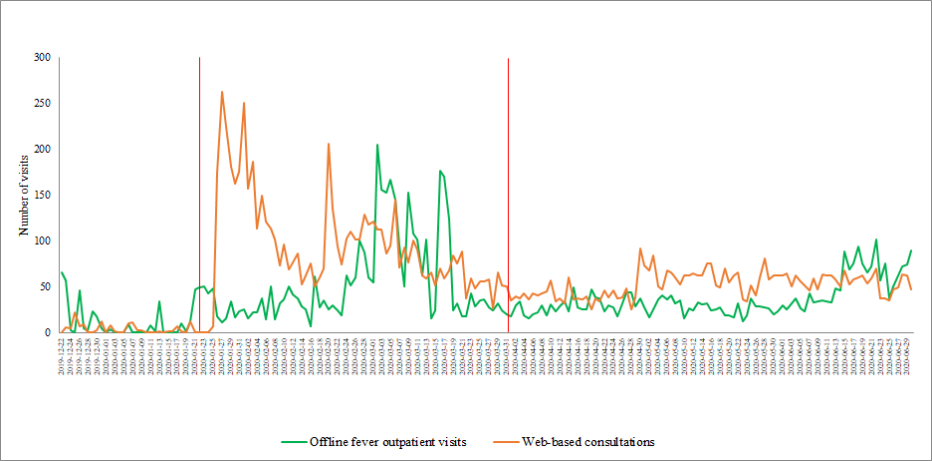
The trends of fever clinic visits and web-based consultation visits from December 22, 2019, to June 30, 2020. The red lines denote the start of the controlled period and the end of the postepidemic period.

Hospital outpatient visits averaged 3266 per day prior to the COVID-19 outbreak, then dropped to approximately 1182 visits per day during the controlled period and slightly increased to about 2699 visits per day in the postepidemic period. Although fever clinic visits increased from 11 per day prior to the outbreak to 56 per day during the controlled period and 37 visits per day in the postepidemic period (Table 1), Figure 5 shows that the outpatient visits dropped significantly early in the outbreak and ascended slowly afterward. The trends of fever clinic visits and web-based consultations significantly increased in the early stages of the COVID-19 outbreak, then decreased to a level that was relatively low but still higher than that in the pre-epidemic period. The fever clinic visits reached a peak on March 2, 2020, 30 days after the peak of web-based consultations (Figure 6).

**Table 1:** Hospital outpatient service volumes and platform service usage data by functionality in the pre-epidemic, controlled, and postepidemic periods.

Functionality	Pre-epidemic period (December 22, 2019, to January 22, 2020)		Controlled period (January 23 to March 31, 2020)		Postepidemic period (April 1 to June 30, 2020)	
	Total uses	Uses per day	Total uses	Uses per day	Total uses	Uses per day
Outpatient visit	104,498	3266	81,561	1182	245,605	2699
Fever clinic visit	356	11	3886	56	3371	37
Automated COVID-19 screening	N/A^a^	N/A	93,405	1354	68,479	752
COVID-19–related symptom monitoring	N/A	N/A	2,006,178	29,075	5,775,357	63,465
General web-based consultation	960	30	8406	122	6656	73
Psychological counseling	N/A	N/A	474	7	162	2
Web-based prescription	830	26	1781	26	1898	21

^a^N/A: not applicable.

The platform service usage data are shown in Table 1. By the end of June 2020, 96,642 people had used the automated COVID-19 screening and symptom monitoring services 161,884 and 7,795,194 times, respectively. The number of general web-based consultation services per day increased from 30 visits in the pre-epidemic period to 122 visits during the controlled period, then dropped to 73 visits in the postepidemic period. The psychological counseling platform served 636 clients during the epidemic.

Of the 161,884 people who used the automated COVID-19 screening service, 160,916 users (99.40%) were classified as at no risk, 464 (0.29%) were classified as medium to high risk, and 12 (0.01%) were recommended to undergo COVID-19 testing and treatment. The results of the automated COVID-19 screening during the controlled period and postepidemic period are shown in Table 2.

**Table 2:** Results of automated COVID-19 screening during the controlled period and postepidemic period (N=161,884).

Result	Screens during controlled period (January 23 to March 31, 2020), n (%)	Screens during postepidemic period (April 1 to June 30, 2020), n (%)	Total screens, n (%)
Screened	93,405 (100)	68,479 (100)	161,884 (100)
No risk	92,704 (99.25)	68,212 (99.61)	160,916 (99.40)
Low risk (home observation)	435 (0.47)	57 (0.08)	492 (0.30)
Medium to high risk (quarantine)	256 (0.27)	208 (0.30)	464 (0.29)
High risk (treatment)	10 (0.01)	2 (0.01)	12 (0.01)

Among the 96,642 individuals who used the COVID-19–related symptom monitoring service, 6696 (6.9%) were symptomatic at some point during monitoring. Fever was the most frequently reported symptom; 2684/6696 symptomatic people (40.1%) had fever. Cough and sore throat were also recurrently reported by symptomatic clients (1657/6696, 24.7%, and 1622/6696, 24.3%, respectively). The results of the COVID-19–related symptom monitoring are shown in Table 3.

**Table 3:** Results of the COVID-19–related symptom monitoring (N=96,642).

Symptom	Controlled period (January 23 to March 31, 2020)		Postepidemic period (April 1 to June 30, 2020)	
	Persons monitored (n=46,456), n (%)	Times monitored	Persons monitored (n=50,186), n (%)	Times monitored
Asymptomatic	43,988 (94.7)	2,006,178	45,958 (91.6)	5,775,357
Symptomatic	2468 (5.3)	5673	4228 (8.4)	10,343
Fever	919 (37.2)	2666	1765 (41.7)	5219
Runny nose	241 (9.8)	382	468 (11.1)	766
Cough	623 (25.2)	1111	1036 (24.5)	1777
Sore throat	565 (22.9)	897	1057 (25.0)	1605
Fatigue	399 (16.2)	617	616 (14.6)	976

## Discussion

### Principal Findings

This article introduces the practical experience of the application of digital health technologies in response to the COVID-19 pandemic from the perspective of a public tertiary hospital in China. The web-based COVID-19 service platform was integrated with automated COVID-19 screening, daily symptom monitoring, web-based care services, and knowledge dissemination to achieve prehospital triage, supplement offline medical care, and facilitate disease control and prevention. Preliminary data show that this practice has good acceptability among the public and sound applicability for complementing hospital services during an emergency crisis. Our practice can serve as a structure model for hospitals to develop their own digital health services that tailor to their technology infrastructure, the disease epidemic characteristics, and the need for disease control.

This web-based COVID-19 service platform features several functionalities that respond to the pain points of disease epidemics [20]. Automated COVID-19 screening realizes prehospital triage for patients before they arrive at the hospital based on epidemiological evidence of COVID-19 using a decision tree algorithm [21]. This work substantially reduces the burden on the fever clinic service and effectively prevents nosocomial infections. Additionally, daily monitoring of COVID-19 symptoms helps administrative and health staff to manage links with web-based medical services, which guarantees a systematic flow of work. Moreover, web-based consultation provides medical services for individuals in a virtual setting, which supplements the suspension of offline medical services. Psychological counseling services provide free, professional, and systematic psychological assistance to users of web-based and offline services to prevent and alleviate the psychological distress caused by the epidemic [22]. Furthermore, this web-based service platform overcomes time and geographical limitations, which enables people to conveniently access professional care services.

The platform service usage data show that the number of web-based consultations sharply increased during the controlled period and then slightly decreased in the postepidemic period; however, this number was still far higher than that during the pre-epidemic period. In contrast, we established that the number of offline outpatient visits dropped significantly in the controlled period. These results suggest the applicability of using web-based medical services to address the challenge of maintaining medical services while reducing the likelihood of nosocomial infection.

### Implications

Our work has several policy, implementation, and research implications. First, from the policy perspective, based on the experience of the COVID-19 pandemic, policy makers should be driven toward developing a contingency plan that includes strategies of promotion and regulation of web-based medical services by defining the scopes and standards as well as the rights and responsibilities of the entities [23]. Secondly, from the implementation perspective, hospitals should plan ahead for the establishment of internet hospitals in accordance with local conditions, establish digital health technologies, and formulate emergency response measures against severe infectious disease outbreaks [24]. However, there is no “one-size-fits-all” strategy for all hospitals; the functionalities should be tailored to each hospital’s needs, and the available tools should be shared within the medical consortium to achieve the highest cost-effectiveness. In addition, from the research perspective, strengthening research on web-based health services, including the scope of diagnosis and care of internet hospitals, acceptability for different subgroup populations, digital health solutions, and quality control measures, is warranted [25].

### Recommendations

Although this paper has demonstrated the capabilities of this system to prevent and fight COVID-19 to a certain extent, there are several ways to further strengthen the system. First, on the premise of ensuring information security, connecting the hospital’s electronic medical record database with internet hospital information can provide patients with more comprehensive and reliable medical care services. Also, customizing medical services for different groups of populations, such as web-based medical visits and home monitoring for chronic patients, can improve the efficiency of disease diagnosis and treatment and the satisfaction of patients [6]. It is also important to note that medical insurance payments for web-based medical services can further increase patients’ willingness to use these services.

### Conclusions

A web-based COVID-19 service platform at a tertiary hospital in China is presented as a role model for using digital health technologies to respond to the COVID-19 pandemic. The digital solutions of automated COVID-19 screening, daily symptom monitoring, web-based care, and knowledge access have commendable acceptability and feasibility for complementing offline hospital services and facilitating disease control and prevention. Future studies to evaluate the effects of relevant functions on practical applications and formulate relevant policies and measures to enhance the application of digital health technologies are of paramount importance.

## Appendix

Multimedia Appendix 1 Definitions of the risk categories in the automated COVID-19 screening service.

## Abbreviations

AI: artificial intelligence
GD2H: Guangdong Second Provincial General Hospital

## References

[1] Ageron F, Sarasin F, Pasquier M, Carron P (2020). Emergency department : COVID-19 crisis and organizational aspects. Article in French. Rev Med Suisse.

[2] Elmore JG, Wang P, Kerr KF, Schriger DL, Morrison DE, Brookmeyer R, Pfeffer MA, Payne TH, Currier JS (2020). Excess Patient Visits for Cough and Pulmonary Disease at a Large US Health System in the Months Prior to the COVID-19 Pandemic: Time-Series Analysis. J Med Internet Res.

[3] He Y, Li W, Wang Z, Chen H, Tian L, Liu D (2020). Nosocomial infection among patients with COVID-19: A retrospective data analysis of 918 cases from a single center in Wuhan, China. Infect Control Hosp Epidemiol.

[4] Huang H, Zhao W, Li G (2020). Knowledge and Psychological Stress Related to COVID-19 Among Nursing Staff in a Hospital in China: Cross-Sectional Survey Study. JMIR Form Res.

[5] Chiappetta S, Sharma AM, Bottino V, Stier C (2020). COVID-19 and the role of chronic inflammation in patients with obesity. Int J Obes (Lond).

[6] Guarino M, Cossiga V, Fiorentino A, Pontillo G, Morisco F (2020). Use of Telemedicine for Chronic Liver Disease at a Single Care Center During the COVID-19 Pandemic: Prospective Observational Study. J Med Internet Res.

[7] Atherly A, Van Den Broek-Altenburg E, Hart V, Gleason K, Carney J (2020). Consumer Reported Care Deferrals Due to the COVID-19 Pandemic, and the Role and Potential of Telemedicine: Cross-Sectional Analysis. JMIR Public Health Surveill.

[8] Tu J, Wang C, Wu S (2015). The internet hospital: an emerging innovation in China. Lancet Glob Health.

[9] Chan AT, Drew DA, Nguyen LH, Joshi AD, Ma W, Guo C, Lo C, Mehta RS, Kwon S, Sikavi DR, Magicheva-Gupta MV, Fatehi ZS, Flynn JJ, Leonardo BM, Albert CM, Andreotti G, Beane-Freeman LE, Balasubramanian BA, Brownstein JS, Bruinsma F, Cowan AN, Deka A, Ernst ME, Figueiredo JC, Franks PW, Gardner CD, Ghobrial IM, Haiman CA, Hall JE, Deming-Halverson SL, Kirpach B, Lacey JV, Marchand LL, Marinac CR, Martinez ME, Milne RL, Murray AM, Nash D, Palmer JR, Patel AV, Rosenberg L, Sandler DP, Sharma SV, Schurman SH, Wilkens LR, Chavarro JE, Eliassen AH, Hart JE, Kang JH, Koenen KC, Kubzansky LD, Mucci LA, Ourselin S, Rich-Edwards JW, Song M, Stampfer MJ, Steves CJ, Willett WC, Wolf J, Spector T, COPE Consortium (2020). The COronavirus Pandemic Epidemiology (COPE) Consortium: A Call to Action. Cancer Epidemiol Biomarkers Prev.

[10] Dong E, Du H, Gardner L (2020). An interactive web-based dashboard to track COVID-19 in real time. Lancet Infect Dis.

[11] Kamel Boulos MN, Geraghty EM (2020). Geographical tracking and mapping of coronavirus disease COVID-19/severe acute respiratory syndrome coronavirus 2 (SARS-CoV-2) epidemic and associated events around the world: how 21st century GIS technologies are supporting the global fight against outbreaks and epidemics. Int J Health Geogr.

[12] Wissel B, Van Camp P J, Kouril M, Weis C, Glauser TA, White PS, Kohane IS, Dexheimer JW (2020). An interactive online dashboard for tracking COVID-19 in U.S. counties, cities, and states in real time. J Am Med Inform Assoc.

[13] Cheng W, Hao C (2020). Case-Initiated COVID-19 Contact Tracing Using Anonymous Notifications. JMIR Mhealth Uhealth.

[14] Adorni F, Prinelli F, Bianchi F, Giacomelli A, Pagani G, Bernacchia D, Rusconi S, Maggi S, Trevisan C, Noale M, Molinaro S, Bastiani L, Fortunato L, Jesuthasan N, Sojic A, Pettenati C, Tavio M, Andreoni M, Mastroianni C, Antonelli Incalzi R, Galli M (2020). Self-Reported Symptoms of SARS-CoV-2 Infection in a Nonhospitalized Population in Italy: Cross-Sectional Study of the EPICOVID19 Web-Based Survey. JMIR Public Health Surveill.

[15] Haodf.com. Webpage in Chinese.

[16] Bao H, Cao B, Xiong Y, Tang W (2020). Digital Media's Role in the COVID-19 Pandemic. JMIR mHealth uHealth.

[17] (2020). Diagnosis and Treatment Protocol for Novel Coronavirus Pneumonia (Trial Version 7). National Health Commission of the People’s Republic of China.

[18] (2020). Guidelines for Investigation and Management of Close Contacts of COVID-19 Cases Training Kit from Chinese Center for Disease Control and Prevention. China Center for Disease Control and Prevention.

[19] (2020). https://www.scmp.com/economy/china-economy/article/3064625/coronavirus-first-chinese-province-declare-top-level. South China Morning Post.

[20] Dzieciatkowski T, Szarpak L, Filipiak KJ, Jaguszewski M, Ladny JR, Smereka J (2020). COVID-19 challenge for modern medicine. Cardiol J.

[21] Feretzakis G, Kalles D, Verykios V (2019). Hiding Decision Tree Rules in Medical Data: A Case Study. Stud Health Technol Inform.

[22] Shen J, Duan H, Zhang B, Wang J, Ji JS, Wang J, Pan L, Wang X, Zhao K, Ying B, Tang S, Zhang J, Liang C, Sun H, Lv Y, Li Y, Li T, Li L, Liu H, Zhang L, Wang L, Shi X (2020). Prevention and control of COVID-19 in public transportation: Experience from China. Environ Pollut.

[23] Ramtekkar U, Bridge JA, Thomas G, Butter E, Reese J, Logan E, Lin S, Axelson D (2020). Pediatric Telebehavioral Health: A Transformational Shift in Care Delivery in the Era of COVID-19. JMIR Ment Health.

[24] Sacco G, Lléonart S, Simon R, Noublanche F, Annweiler C, TOVID Study Group (2020). Communication Technology Preferences of Hospitalized and Institutionalized Frail Older Adults During COVID-19 Confinement: Cross-Sectional Survey Study. JMIR Mhealth Uhealth.

[25] Mani VR, Kalabin A, Valdivieso SC, Murray-Ramcharan M, Donaldson B (2020). New York Inner City Hospital COVID-19 Experience and Current Data: Retrospective Analysis at the Epicenter of the American Coronavirus Outbreak. J Med Internet Res.

