# Supplementary material for: Digital Health Technologies Respond to the COVID-19 Pandemic In a Tertiary Hospital in China: Development and Usability Study
Source: J Med Internet Res. 2020 Nov 24;22(11):e24505. doi: 10.2196/24505 (PMC7690252; doi:10.2196/24505)
Supplement: Multimedia Appendix 1 [file jmir_v22i11e24505_app1.docx]

**Supplementary information**

The definitions of risk category in COVID-19 automated screening.

| **Patient information** | **Risk category** | **Recommendation** |
| --- | --- | --- |
| (Fever/ sore throat/ cough/ stuffy nose/ runny nose/ chest tightness/ shortness of breath/ dyspnea/ general aching/ diarrhea) & (Travelled or lived in Wuhan (Hubei)/ Contacted with personnel from Wuhan (Hubei), Wenzhou or Taizhou/ Contacted with confirmed or suspected patients/ Was around people with fever, fatigue, cough, sore throat and so on) | High risk (epidemiological suspect) | See the fever clinic immediately |
| (Fever/ sore throat/ cough/ stuffy nose/ runny nose/ chest tightness/ shortness of breath/ dyspnea/ general aching/ diarrhea) & no contact history | Median to High risk | Close contacts should be isolated at home for at least 2 weeks |
| No typical symptoms & (Travelled or lived in Wuhan (Hubei)/ Contacted with personnel from Wuhan (Hubei) / Contacted with confirmed or suspected patients/ Was around people with fever, fatigue, cough, sore throat and so on) | Low risk | Continue to monitor body temperature at home |
| No typical symptoms & No contact history | No risk | Wear masks, wash hands frequently, avoid crowd gathering, and reduce unnecessary going out |

Note: Information was translated from the Judgment Logic Explanation of Intelligent Assisted COVID-19 Questionnaire, which recommended by National Health Commission of the P.R.C. (<http://www.nhc.gov.cn/yzygj/s7653p/202002/ec5e345814e744398c2adef17b657fb8.shtml>, access on September 25, 2020)
